# Supplementary figures and images for: The chromatin remodeller CHD4 regulates transcription factor binding to both prevent activation of silent enhancers and maintain active regulatory elements
Source: eLife. 2026 Feb 3;14:RP109280. doi: 10.7554/eLife.109280 (PMC12867480; doi:10.7554/eLife.109280)

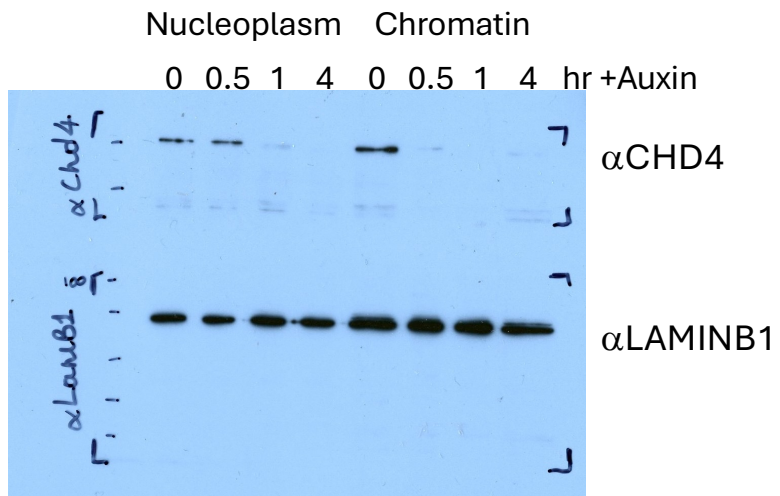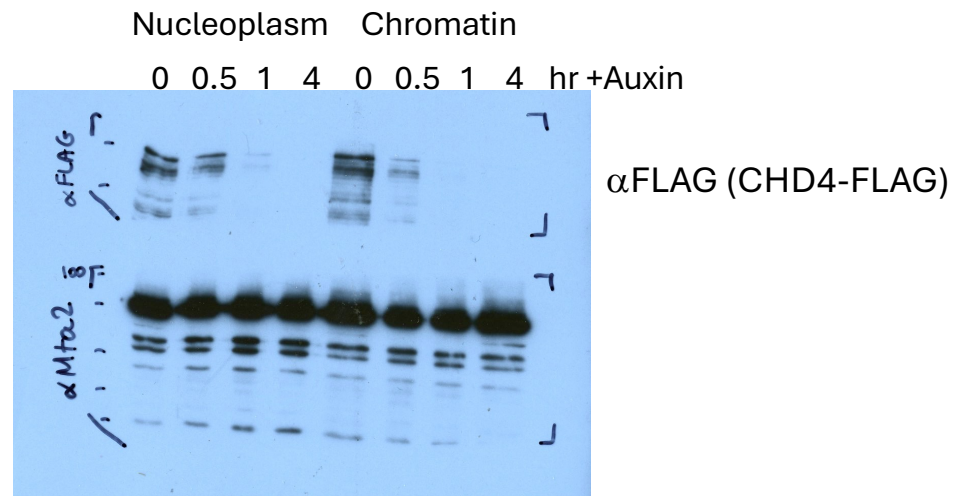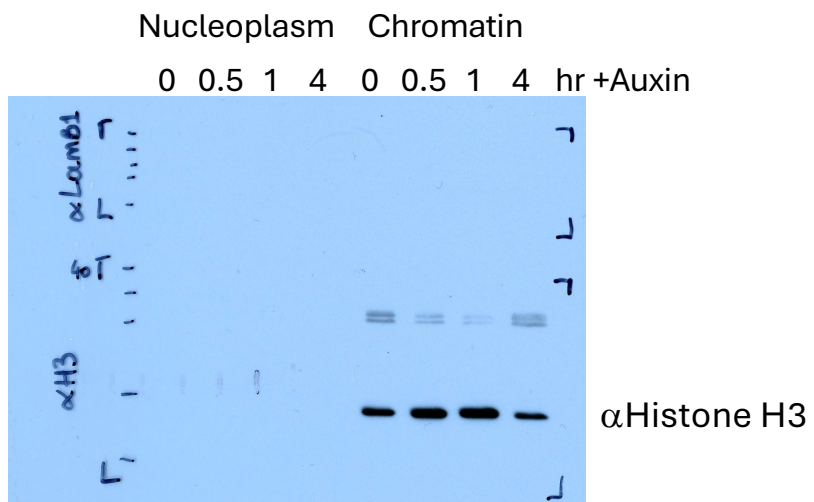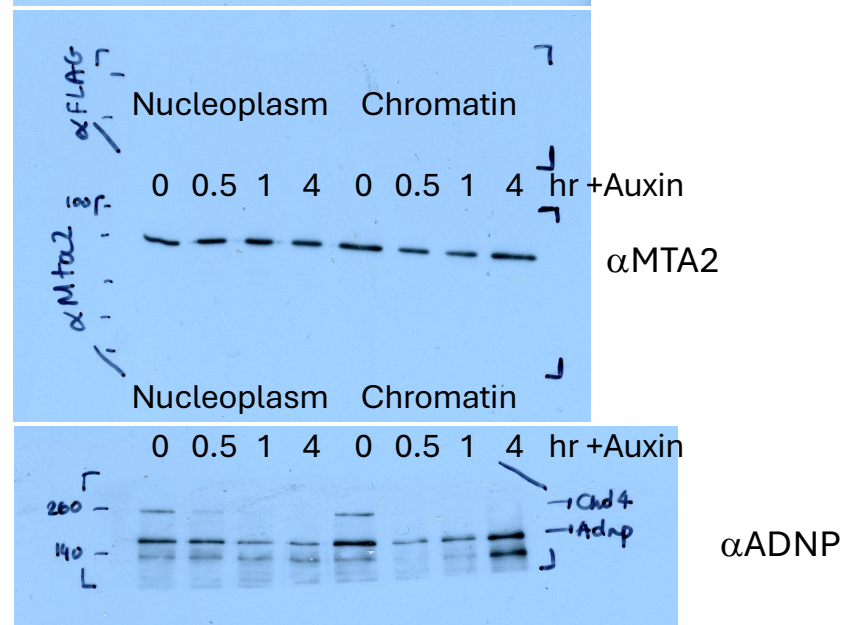

Supplement: Figure 1—source data 1. [file elife-109280-fig1-data1.zip › Figure 1 - Source Data 1.pdf]

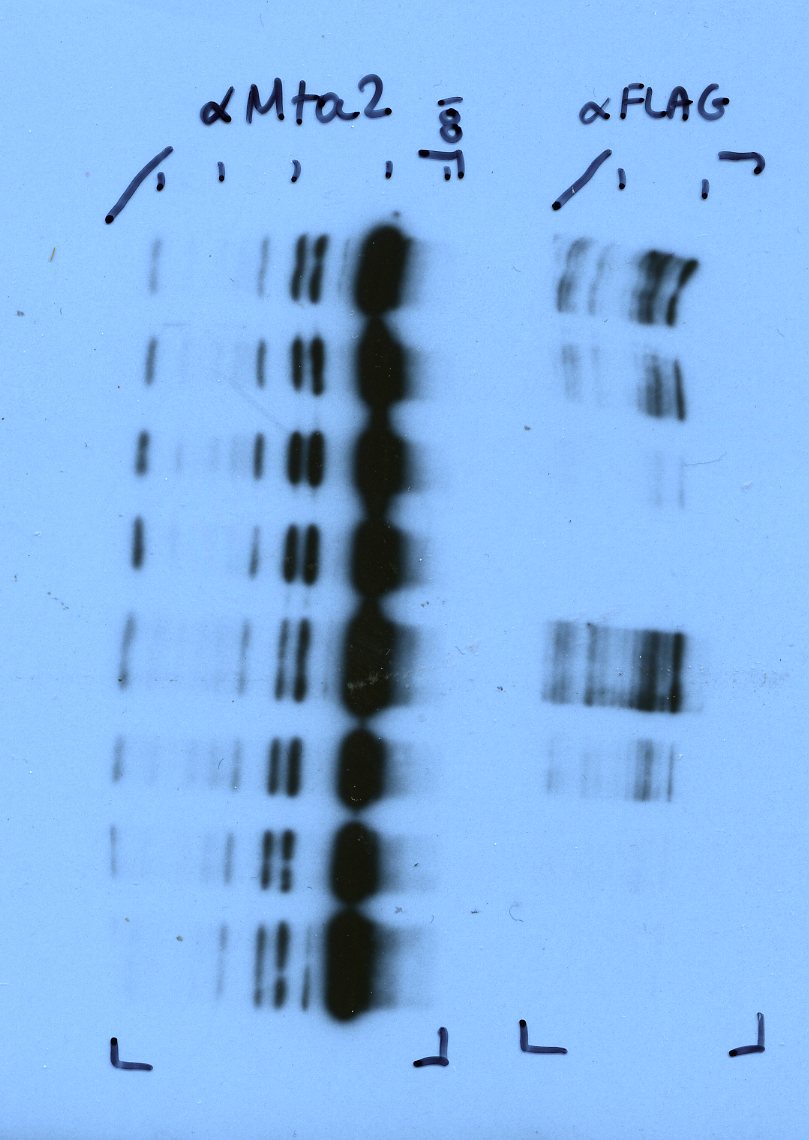

Supplement: Figure 1—source data 2. [file elife-109280-fig1-data2.zip › Figure 1 _Source Data 2/Nuc_Chr_FLAG_Mta2001.jpg]

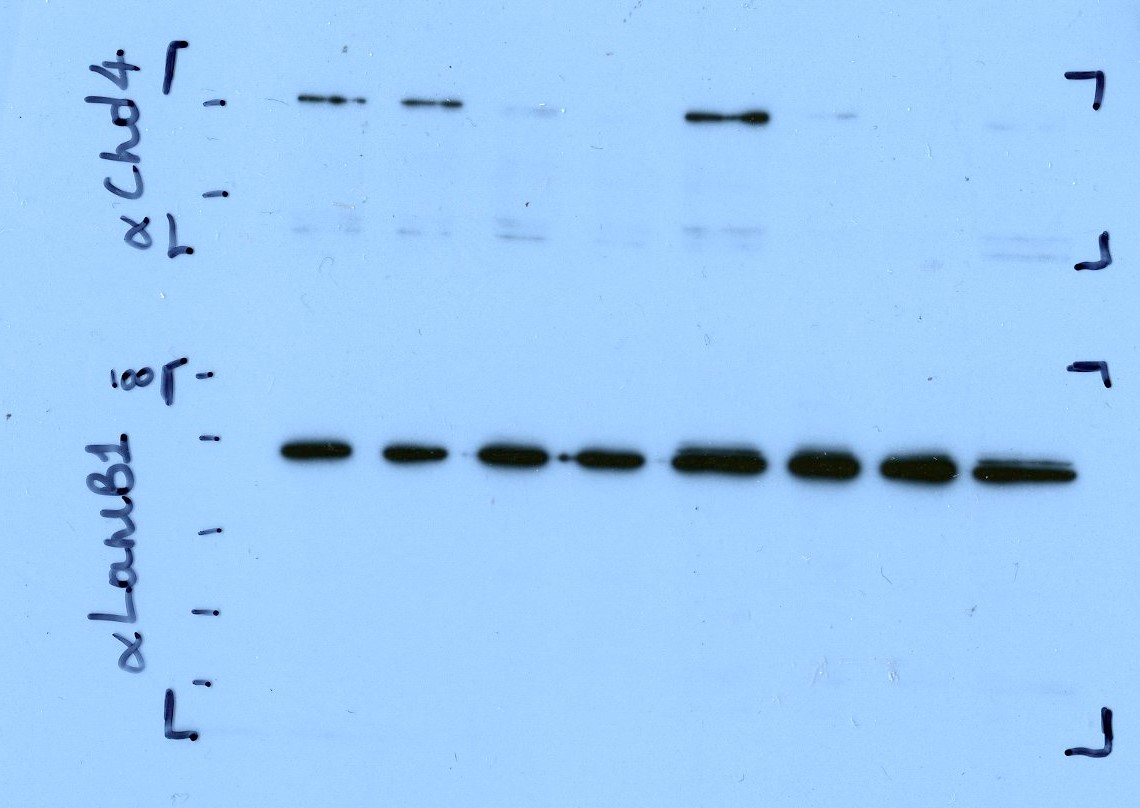

Supplement: Figure 1—source data 2. [file elife-109280-fig1-data2.zip › Figure 1 _Source Data 2/Nuc_Chr_Chd4_Lamb1005.jpg]

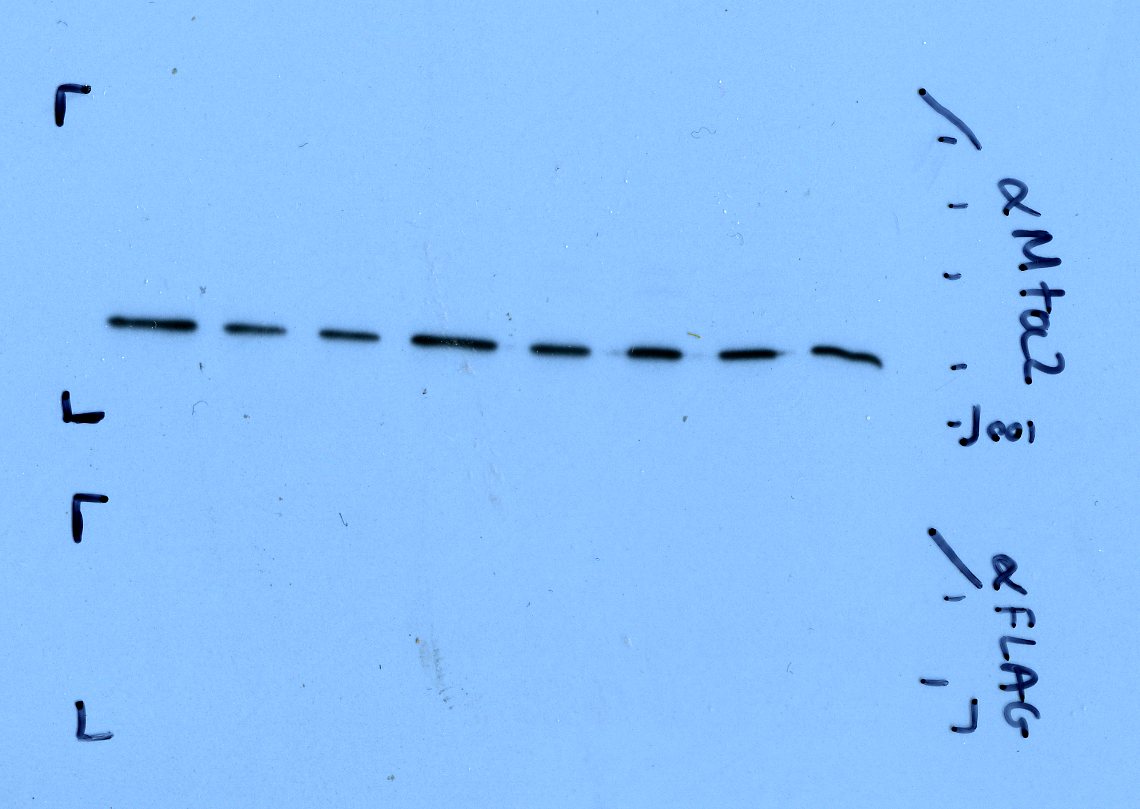

Supplement: Figure 1—source data 2. [file elife-109280-fig1-data2.zip › Figure 1 _Source Data 2/Nuc_Chr_FLAG_Mta2006.jpg]

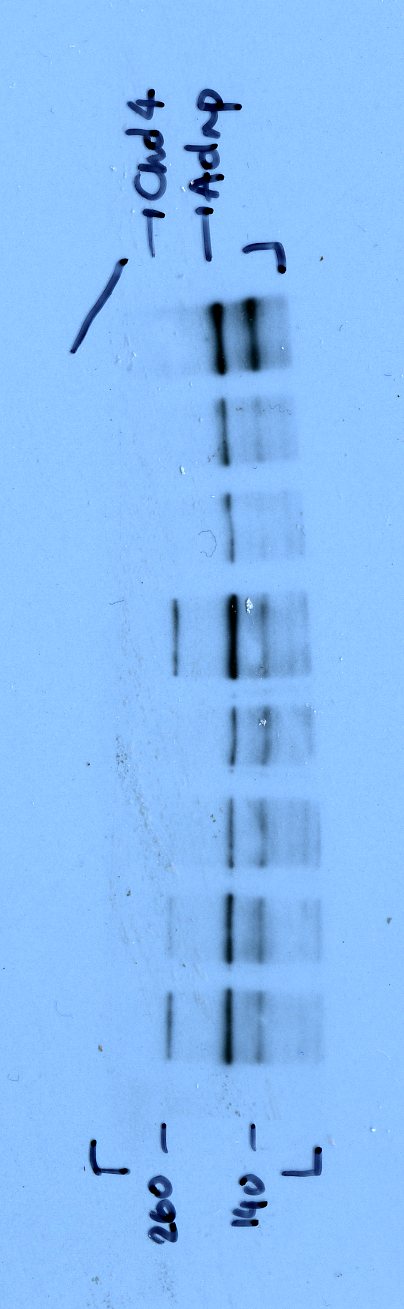

Supplement: Figure 1—source data 2. [file elife-109280-fig1-data2.zip › Figure 1 _Source Data 2/Nuc_Chr_Chd4_Adnp003.jpg]

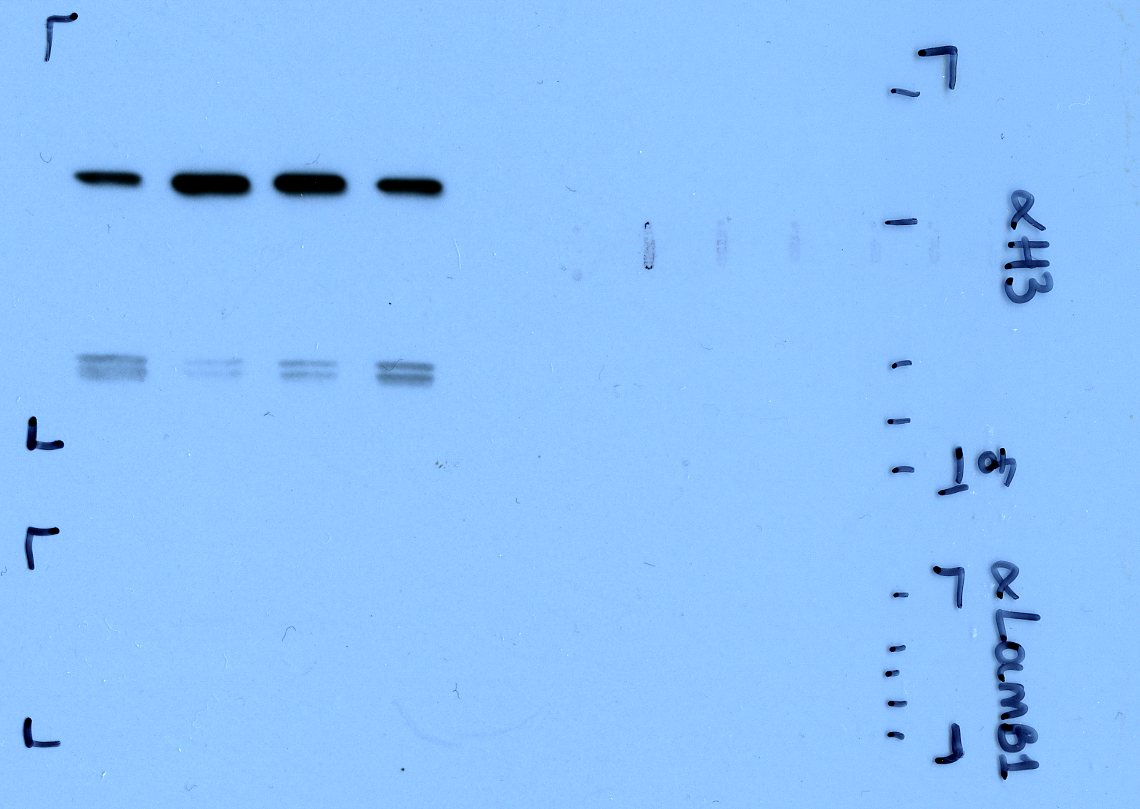

Supplement: Figure 1—source data 2. [file elife-109280-fig1-data2.zip › Figure 1 _Source Data 2/Nuc_Chr_H3_Lamb1004.jpg]

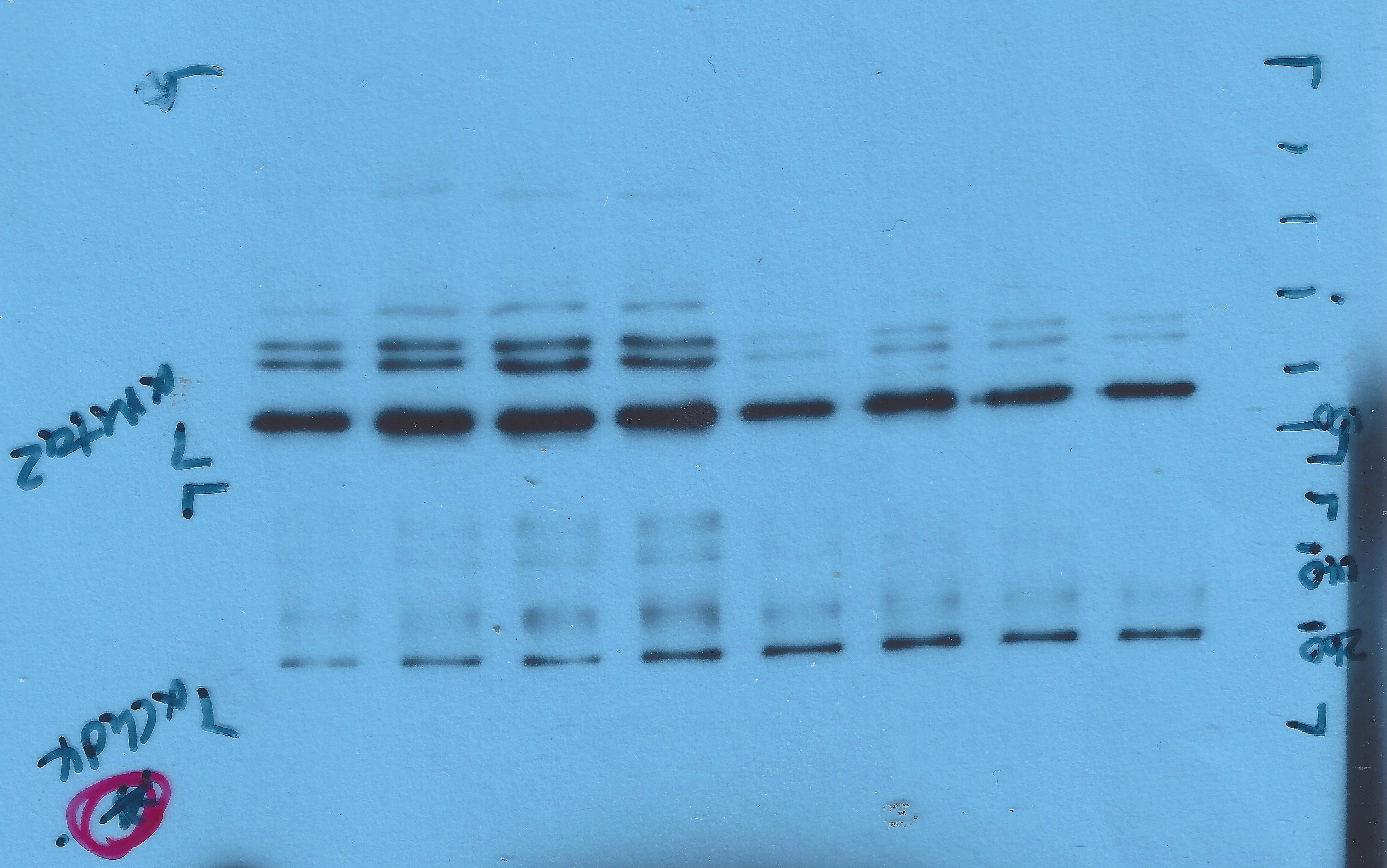

Supplement: Figure 5—source data 2. [file elife-109280-fig5-data2.zip › Figure 5_Source Data 2/CHD4_MTA2longxpo.jpg]

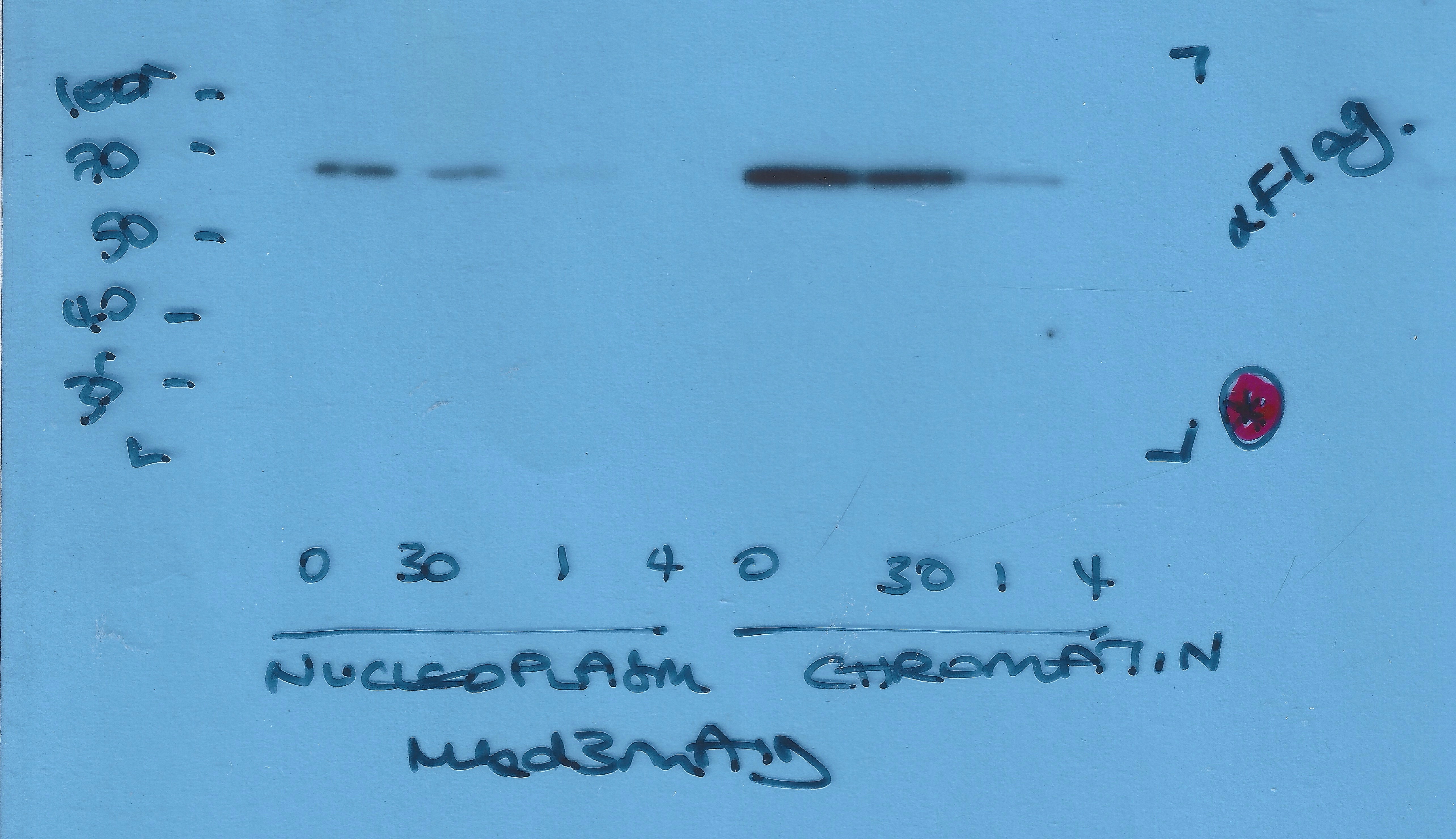

Supplement: Figure 5—source data 2. [file elife-109280-fig5-data2.zip › Figure 5_Source Data 2/FLAG.jpg]

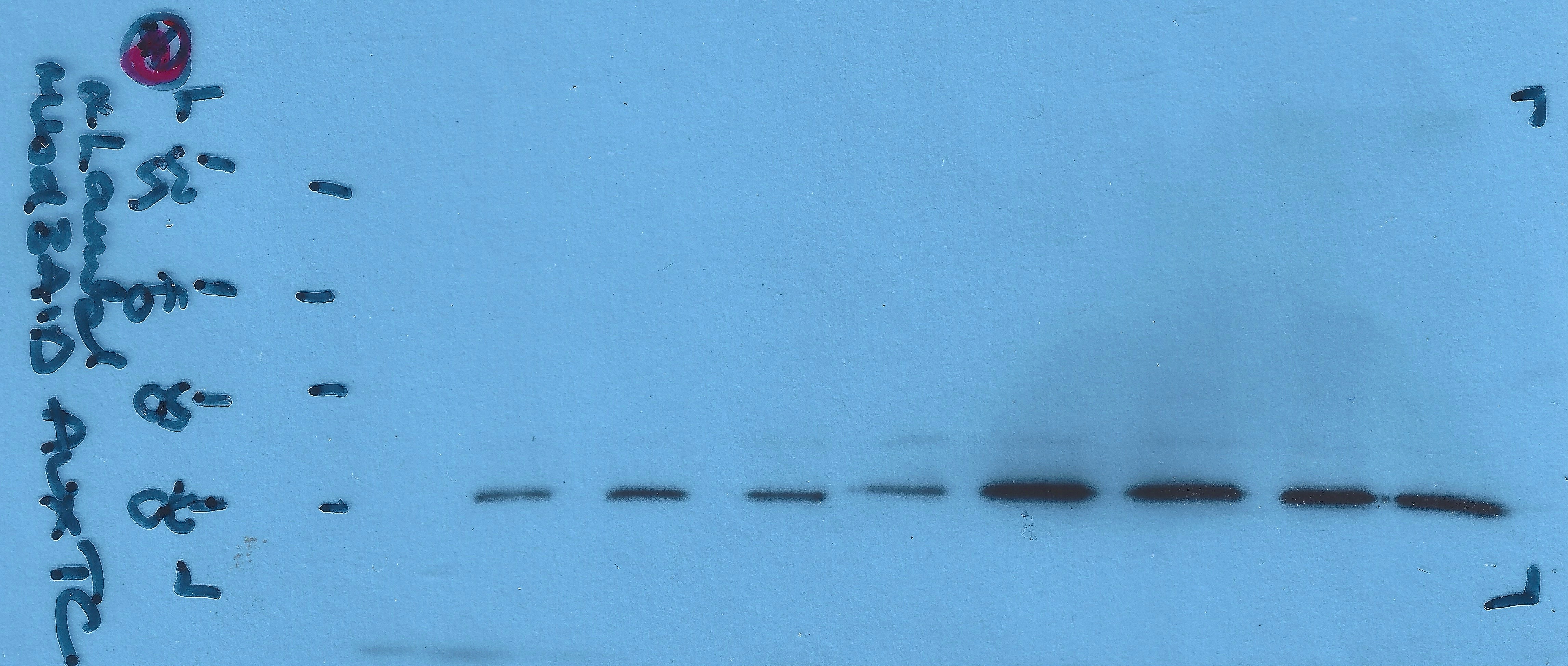

Supplement: Figure 5—source data 2. [file elife-109280-fig5-data2.zip › Figure 5_Source Data 2/LamB1.jpg]

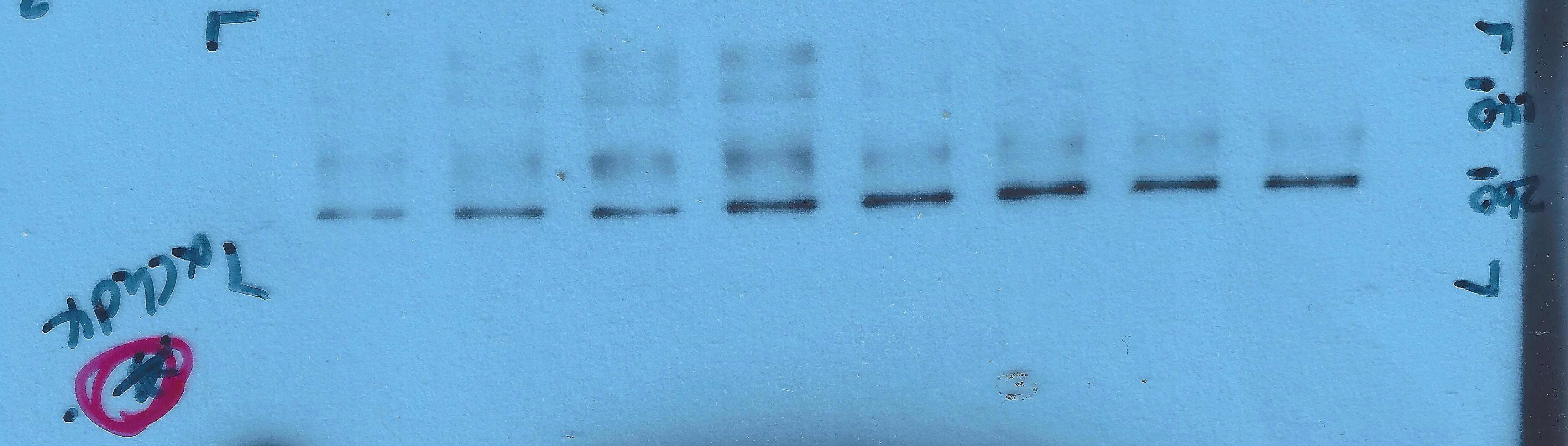

Supplement: Figure 5—source data 2. [file elife-109280-fig5-data2.zip › Figure 5_Source Data 2/CHD4.jpg]

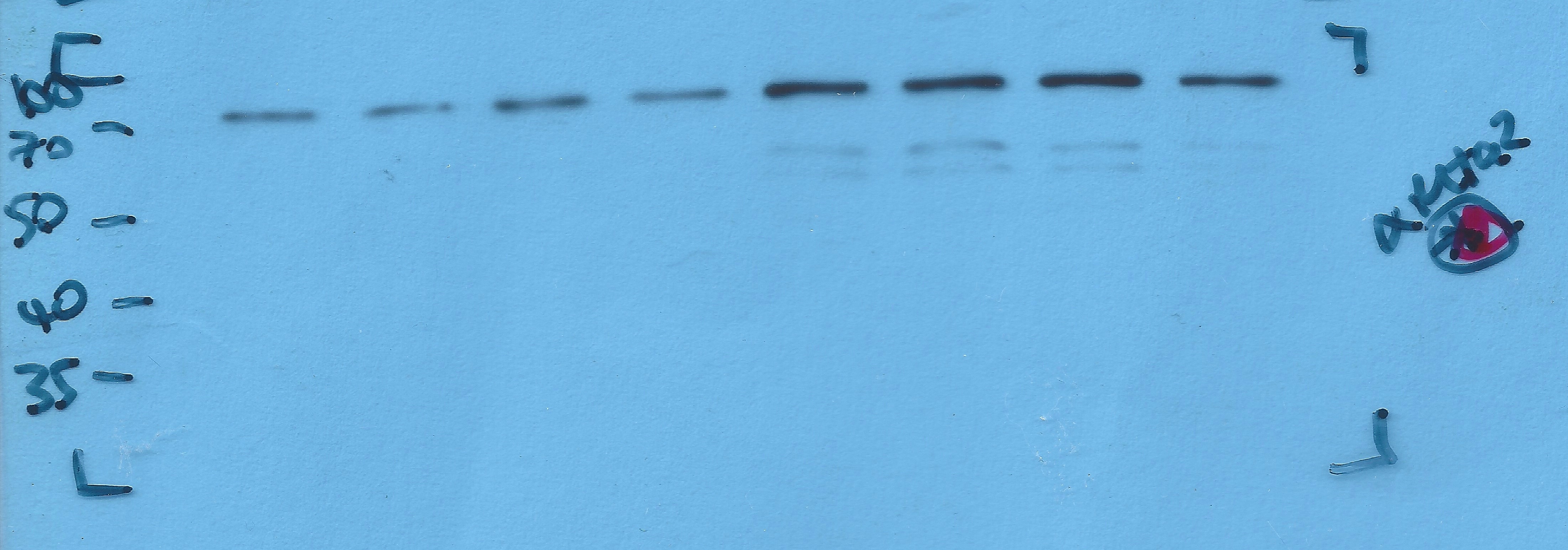

Supplement: Figure 5—source data 2. [file elife-109280-fig5-data2.zip › Figure 5_Source Data 2/MTA2.jpg]

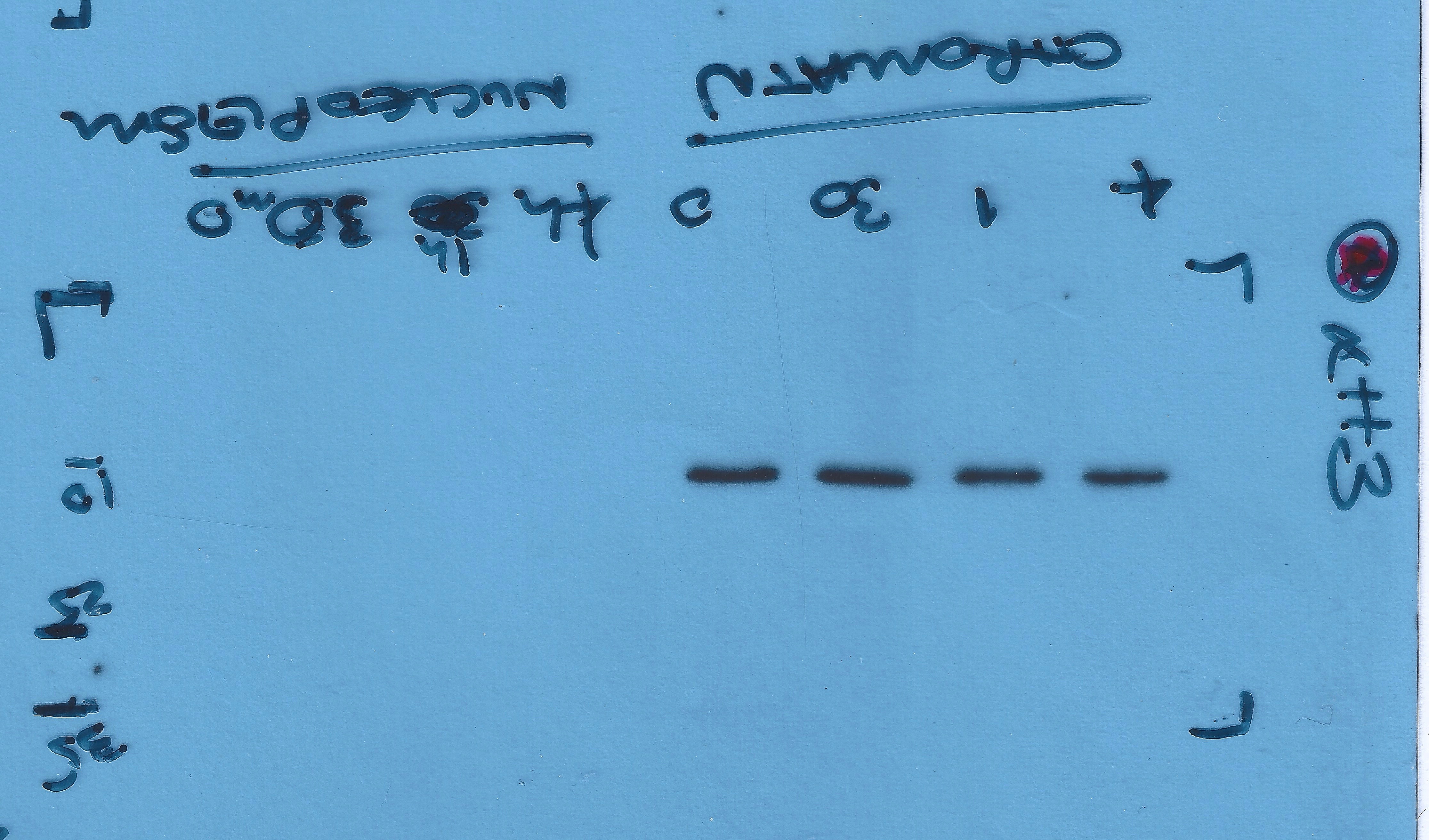

Supplement: Figure 5—source data 2. [file elife-109280-fig5-data2.zip › Figure 5_Source Data 2/H3.jpg]
