# Supplementary material for: The chromatin remodeller CHD4 regulates transcription factor binding to both prevent activation of silent enhancers and maintain active regulatory elements
Source: eLife. 2026 Feb 3;14:RP109280. doi: 10.7554/eLife.109280 (PMC12867480; doi:10.7554/eLife.109280)
Supplement: Figure 5—source data 1. [file elife-109280-fig5-data1.zip › Figure 5_Source Data 1.pdf]

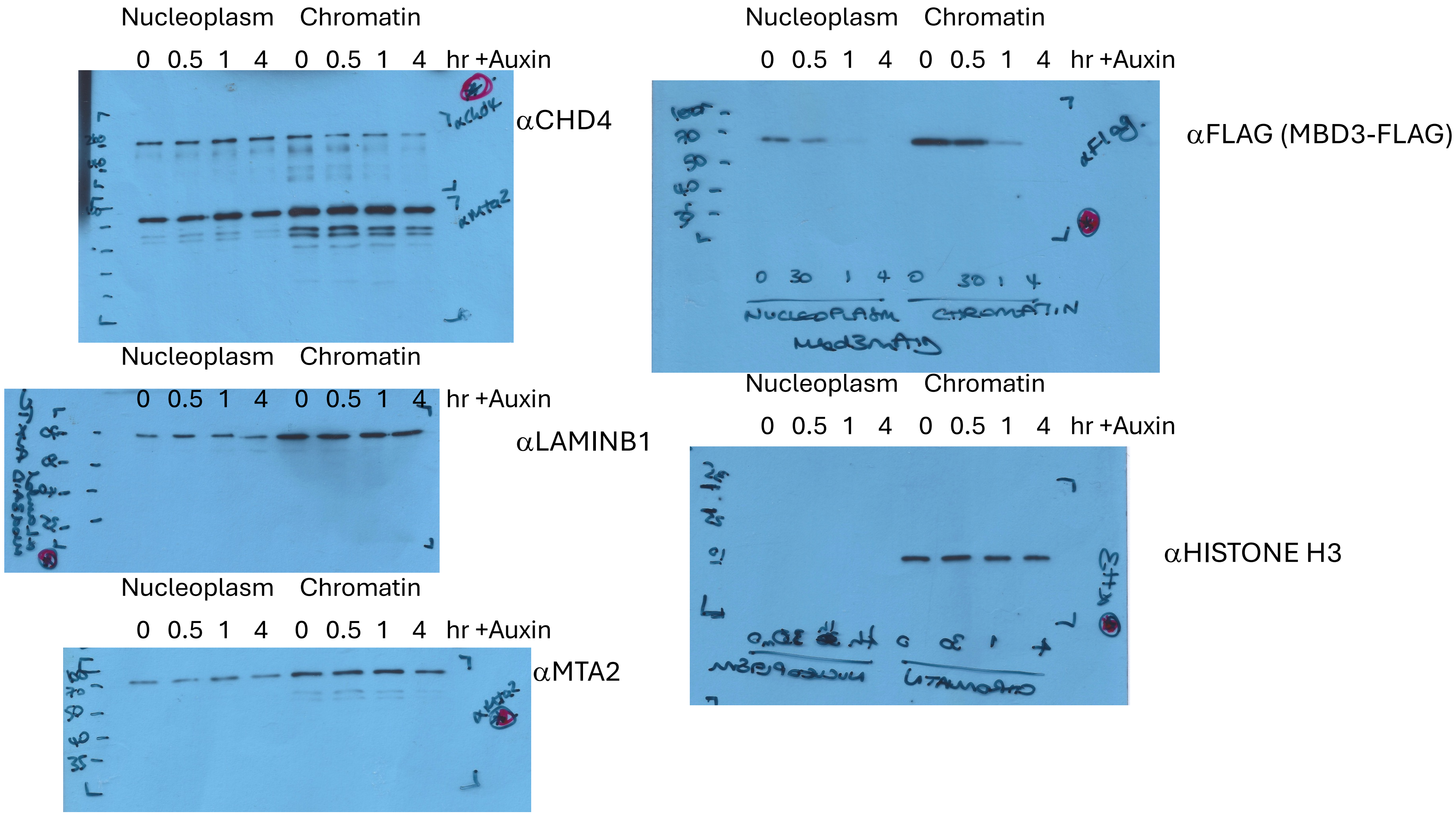
Nucleoplasm Chromatin
0 0.5 1 4 0 0.5 1 4 hr +Auxin
αCHD4
Nucleoplasm Chromatin
0 0.5 1 4 0 0.5 1 4 hr +Auxin
αLAMINB1
Nucleoplasm Chromatin
0 0.5 1 4 0 0.5 1 4 hr +Auxin
αMTA2
Nucleoplasm Chromatin
0 0.5 1 4 0 0.5 1 4 hr +Auxin
αFLAG (MBD3-FLAG)
Nucleoplasm Chromatin
0 0.5 1 4 0 0.5 1 4 hr +Auxin
αHISTONE H3
